# Integrative transcriptomic profiling of mRNA, miRNA, circRNA, and lncRNA in alveolar macrophages isolated from PRRSV-infected porcine

**DOI:** 10.3389/fimmu.2023.1258778

**Published:** 2023-08-24

**Authors:** Ouyang Peng, Yu Xia, Ying Wei, Siying Zeng, Chuangchao Zou, Fangyu Hu, Qiuping Xu, Yihui Huang, Rui Geng, Guangli Hu, Yongchang Cao, Hao Zhang

**Affiliations:** ^1^ State Key Laboratory of Biocontrol, School of Life Sciences, Sun Yat-sen University, Guangzhou, China; ^2^ College of Animal Science and Technology/Luoyang Key Laboratory of Live Carrier Biomaterial and Animal Disease Prevention and Control, Henan University of Science and Technology, Luoyang, China; ^3^ Guangdong Provincial Key Laboratory of Malignant Tumor Epigenetics and Gene Regulation, Sun Yat-sen Memorial Hospital, Sun Yat-sen University, Guangzhou, China

**Keywords:** porcine reproductive and respiratory syndrome virus, integrative transcriptomic profiling, functional enrichment analysis, immune response, circular RNA, ceRNA network

## Abstract

**Introduction:**

The porcine reproductive and respiratory syndrome virus (PRRSV) continues to pose a significant threat to the global swine industry, attributed largely to its immunosuppressive properties and the chronic nature of its infection. The absence of effective vaccines and therapeutics amplifies the urgency to deepen our comprehension of PRRSV’s intricate pathogenic mechanisms. Previous transcriptomic studies, although informative, are partially constrained by their predominant reliance on in vitro models or lack of long-term infections. Moreover, the role of circular RNAs (circRNAs) during PRRSV invasion is yet to be elucidated.

**Methods:**

In this study, we employed an in vivo approach, exposing piglets to a PRRSV challenge over varied durations of 3, 7, or 21 days. Subsequently, porcine alveolar macrophages were isolated for a comprehensive transcriptomic investigation, examining the expression patterns of mRNAs, miRNAs, circRNAs, and long non-coding RNAs (lncRNAs).

**Results:**

Differentially expressed RNAs from all four categories were identified, underscoring the dynamic interplay among these RNA species during PRRSV infection. Functional enrichment analyses indicate that these differentially expressed RNAs, as well as their target genes, play a pivotal role in immune related pathways. For the first time, we integrated circRNAs into the lncRNA-miRNA-mRNA relationship, constructing a competitive endogenous RNA (ceRNA) network. Our findings highlight the immune-related genes, CTLA4 and SAMHD1, as well as their associated miRNAs, lncRNAs, and circRNAs, suggesting potential therapeutic targets for PRRS. Importantly, we corroborated the expression patterns of selected RNAs through RT-qPCR, ensuring consistency with our transcriptomic sequencing data.

**Discussion:**

This study sheds lights on the intricate RNA interplay during PRRSV infection and provides a solid foundation for future therapeutic strategizing.

## Introduction

Porcine reproductive and respiratory syndrome (PRRS) has emerged as one of the most economically impactful porcine diseases over the past three decades (1). It poses a significant and ongoing threat to the global swine industry due to its induction of respiratory distress in piglets, and its causation of stillbirths, mummified fetuses, and abortions in gestating sows, leading to substantial economic losses (2, 3). PRRS is attributed to the porcine reproductive and respiratory syndrome virus (PRRSV), a member of *Arteriviridae* family within the *Nidovirales* order. This virus is characterized as a single-stranded positive-sense RNA virus with an approximate genome size of 15kb (4, 5). PRRSV demonstrates strict tropism and is capable of proliferating within porcine alveolar macrophages (PAMs), the primary target cells in vivo, and inducing an immunosuppression (6). Base on phylogenetical analysis, PRRSVs are primarily categorized into two genotypes: European-like (PRRSV-1) isolates and North American-like (PRRSV-2) isolates, which shared approximately 60% nucleotide sequence identity and were identified in 1991 in Europe and 1992 in North America, respectively (7). The formidable challenges of developing effective vaccines and drugs against PRRSV are heightened by the virus’s rapid mutation rate (8–10), immunosuppressive nature (11, 12), propensity for persistent infection (13, 14), vertical transmission capability (13) and phenomena of antibody-dependent enhancement (ADE) (15). Therefore, the pressing need for a comprehensive and in-depth investigation of PRRSV’s pathogenic mechanisms becomes clear, and this understanding will lay a solid foundation for the development of more effective preventive and therapeutic measures against PRRS.

Within the cellular environment, the repertoire of RNA extends beyond protein-coding messenger RNAs (mRNAs) to include non-coding RNAs (ncRNAs) such as microRNAs (miRNAs), circular noncoding RNAs (circRNAs) and long noncoding RNAs (lncRNAs) (16, 17). These diverse RNA species serve as central players in the genesis and progression of various pathologies, including cancer and viral diseases (18, 19). MiRNAs, spanning approximately 17-25 nucleotides in length, exert influence on gene expression by binding specifically to the 3’ untranslated regions (UTR) of target mRNAs, thus inhibiting gene expression. For instance, during the progression of Chandipura virus (CHPV) infection, the upregulated expression of miR-155 suppresses the suppressor of cytokine signaling 1 (SOCS1), culminating in enhanced phosphorylation of signal transducer and activator of transcription 1 (STAT1) and increased production of interferon-β (IFN-β) (20). LncRNAs, exceeding 200 nucleotides, also engage in cellular signaling pathways, interacting in either miRNA-dependent or independent manner. Viral invasions instigate host-driven lncRNAs that participate in innate immune responses, consequently modulating the dynamic interplay between the host and the virus (21, 22). In the context of hepatitis B virus (HBV) infection, lncRNA n335586 potentiates the expression of host gene CKMT1A by competitively banding miR-924, thereby inducing the migration and invasion of hepatocellular carcinoma (HCC) cells (22). CircRNAs, defined by their covalently closed loop structure, are recognized as crucial regulators in the host’s antiviral defense, functioning via both miRNA-dependent and independent mechanisms (23, 24). Their inherent stability and immunogenicity render circRNAs potent candidates for therapeutic targets or biomarkers (25). As an exemplar, circRNA-0050463 is documented to operate as a sponge for miR-33-5b, thereby controlling EEF1A1 and fostering the proliferation of influenza A virus H1N1 (24). These advancements further highlight the intricate complexities and critical roles of diverse RNA species in cellular signaling, regulation, and host-pathogen interactions. Despite these insights, a significant gap remains in our understanding, with no research elucidating the role of circRNAs in PRRSV infection reported at the time of this study.

Transcriptomic investigations encompassing both gene expression and noncoding RNAs have significantly propelled our comprehension of PRRSV’s pathogenesis and the interplay between PRRSV and pigs (13, 26–32). However, these studies are frequently limited by their reliance on in vitro models, which inherently fall short of capturing the complexity of the disease course, or lack relevance to persistent infections. For instance, previous research employed transcriptomics to examine PAMs infected with PRRSV for a brief 24-hour period. This study discovered that lnc_000397 impedes PRRSV replication by stimulating the expression of interferon-stimulated genes (ISGs) (33). Moreover, lncRNAs were identified as participants in PRRSV infection by orchestrating the regulation of GPER1 and apoptosis-related genes (13). Further study extended investigations to lncRNAs, miRNAs, and mRNAs of porcine endometrial epithelial cells post-PRRSV infection, unearthing numerous miRNAs and lncRNAs involved in various pathways such as cell apoptosis and p53 signaling (27). In addition, miR-181 has been reported to strongly inhibit PRRSV proliferation through binding the downstream region of open reading frame 4 (ORF4) (34). Furthermore, PRRSV induced miR-142-5p was found to promote virus replication by manipulate ER-phagy via targeting the 3’UTR of gene *Fam134b* (35). While these studies have undoubtedly made valuable contributions to our understanding of interaction between host and PRRSV, a comprehensive insight into the interactions amongst circRNAs, lncRNAs, miRNAs, and mRNAs in the regulatory network post-PRRSV infection in PAMs remains elusive. Such integrative, long-term transcriptomic research in vivo is integral to fully elucidating the complex dynamics of RNA regulation following PRRSV infection and deserves further intensive exploration.

In this study, we uniquely utilized an in vivo model, extending the observation to 21 days to mimic the chronicity of PRRSV infections. Through an extended observation and integrated transcriptomic exploration of PRRSV infection, we identified various differentially expressed RNAs and, for the first time, constructed a ceRNA network inclusive of circRNA. This research may shed light on PRRSV pathogenesis and opens avenues for future therapeutic interventions.

## Materials and method

### Experimental design

The experimental subjects for this study comprised of eighteen weaned piglets, aged four weeks. These piglets were chosen based on their negative test results for PRRSV, PEDV, TGEG, PDCoV, SADS-CoV, and PoRV, ensuring they were in good health and had comparable body weights. The piglets were arbitrarily segregated into two groups, each containing 9 individuals. Each group was housed separately in designated areas of the animal facility, and they were granted ad libitum access to food and water for a duration of seven days before the commencement of the study. Prior to the viral challenge, the piglets underwent a fasting period of twelve hours without access to food or water. On the experiment’s initiation day (Day 0), the experimental group was subjected to a dual challenge of PRRSV strain Li11 (6 × 10^7^ TCID_50_) propagated in our laboratory as described previously (36). The control group received an equivalent volume of DMEM. Subsequent to the infection, on 3, 7, and 21 dpi respectively, three piglets from each group were chosen randomly for humane euthanization, and the lung tissues were utilized for PAMs isolation and preserved at -80°C for subsequent use.

### RNA extraction, quantification, and qualification

Total RNA was isolated from PAMs utilizing the TRIzol method. Subsequently, the RNA was examined for degradation and contamination on 1% agarose gels. The NanoPhotometer spectrophotometer (IMPLEN, CA, USA) was employed to assess RNA purity. The RNA concentration was determined using the Qubit RNA Assay Kit in conjunction with the Qubit 2.0 Fluorometer (Life Technologies, CA, USA). Furthermore, the integrity of the RNA was appraised using the RNA Nano 6000 Assay Kit in conjunction with the Agilent Bioanalyzer 2100 system (Agilent Technologies, CA, USA).

### Library preparation for mRNA and lncRNA sequencing

We prepared the RNA library for mRNA and lncRNA sequencing using a rRNA depletion and stranded method. In brief, ribosomal RNA was eradicated from total RNA utilizing the rRNA Removal Kit, in accordance with the manufacturer’s protocol. The RNA was then fragmented into segments of 250-300 bp, and the first-strand cDNA was reverse transcribed from the fragmented RNA using dNTPs. The RNA was then degraded with RNase H, and second-strand cDNA was synthesized using DNA polymerase I and dNTPs. Any remaining overhangs of double-strand cDNA were converted into blunt ends via exonuclease or polymerase activities. Following the adenylation of DNA fragment 3’ ends, sequencing adaptors were ligated to the cDNA. The library fragments were purified with the AMPure XP system to preferentially select cDNA fragments of 250-300 bp in length. Uridine digestion was conducted using Uracil-N-Glycosylase, followed by cDNA amplification via PCR. After library construction, the library concentration was measured using the Qubit fluorometer and adjusted to 1 ng/uL. The Agilent 2100 Bioanalyzer was employed to examine the insert size of the resulting library. Finally, the accurate concentration of the cDNA library was confirmed using qPCR. Provided that the insert size and concentration of the library were consistent, the samples could then be subjected to sequencing.

### Library preparation for small RNA sequencing

A total of 3 μg of total RNA per sample was used as input material for the small RNA library. Sequencing libraries were generated using the NEBNext Multiplex Small RNA Library Prep Set for Illumina (NEB, USA), in line with manufacturer’s guidelines, and index codes were added to attribute sequences to individual samples. In brief, the NEB 3’ SR Adaptor was directly and specifically ligated to the 3’ end of miRNA, siRNA, and piRNA. After the 3’ ligation reaction, the SR RT Primer hybridized to the excess of 3’ SR Adaptor and transformed the single-stranded DNA adaptor into a double-stranded DNA molecule. This step was crucial in preventing adaptor-dimer formation, as dsDNAs are not substrates for ligation mediated by T4 RNA Ligase 1 and therefore do not ligate to the 5’ SR Adaptor in the subsequent ligation step. The 5’ ends adapter was ligated to the 5’ ends of miRNAs, siRNA, and piRNA. Following this, the first-strand cDNA was synthesized using M-MuLV Reverse Transcriptase. PCR amplification was performed using LongAmp Taq 2X Master Mix, SR Primer for Illumina and index (X) primer. PCR products were purified on an 8% polyacrylamide gel. DNA fragments corresponding to 140-160 bp were recovered and dissolved in 8 μL elution buffer. Lastly, the library quality was assessed on the Agilent Bioanalyzer 2100 system using DNA High Sensitivity Chips.

### CircRNA sequencing and identification

RNA was extracted using TRIzol and a minimum of 4 μg of the resulting RNA for each sample was used for circRNA library preparation. Ribosomal RNA was removed from the RNA samples using an Epicentre Ribo-zero rRNA Removal Kit (Epicentre, Madison, WI, USA) to obtain rRNA-depleted RNAs. The rRNA-depleted RNAs were further treated with RNase R (Epicentre) and then subjected to another round of TRIzol extraction. Subsequently, the rRNA-depleted and RNase R-digested RNAs were used to construct sequencing libraries using an NEBNext Ultra Directional RNA Library Prep Kit for Illumina (NEB, Ipswich, MA, USA), according to the manufacturer’s instructions. The remaining procedures were similar to those used for mRNA and lncRNA sequencing. The circRNAs were detected and identified using the CIRI2 (v2.0.6) software (37).

### Sequencing

Upon completion of library preparation and sample pooling, the samples were submitted for sequencing on the Illumina NovaSeq 6000 platform. For mRNA, lncRNA, and circRNA, a PE150 (paired-end 150 nt) sequencing strategy was employed, while for miRNA, a SE150 (single-end 150 nt) strategy was utilized.

### Quality control for raw data

Raw data, in FASTQ format, were initially processed using fastQC software (v0.11.9) available at (http://www.bioinformatics.babraham.ac.uk/projects/fastqc/). We first removed reads with 5’ adapters, followed by those lacking a 3’ adapter or insert sequence. To maintain data quality, we eliminated reads with more than 10% N bases and those with over 50% of bases having a Qphred score of <=20. Reads with poly A/T/G/C sequences were also excluded. Concurrently, adapter sequences from the 3’ ends of reads were trimmed. Quality indicators, including Q20, Q30, and GC content of the refined data, were computed. All downstream analyses were grounded on this high-quality data.

### Reads mapping and transcriptome assembly

An index of the Sus scrofa reference genome assembly (Sus Scrofa v11.1) was constructed, and paired-end clean reads were aligned to the reference genome using HISAT2 (v2.0.4) (38). HISAT2 was operated with ‘–rna-strandness RF’, with all other parameters set to default. Each sample’s mapped reads were assembled using StringTie (v1.3.3) (39) in a reference-based approach.

### Differential expression analysis

Gene expression levels were estimated by fragments per kilobase per million (FPKM) values, obtained using Cuffdiff (v2.0.1) (40), and transcripts with an absolute log2-fold change of ≥ 1 and an adjusted p-adjust value of ≤ 0.05 were identified as differentially expressed.

### Target RNA prediction

The target mRNAs of differentially expressed miRNAs, the target miRNAs of circRNAs, or the target miRNAs of differentially expressed lncRNAs were predicted using three software tools: TargetScan (v7.2) (41), miRanda (v2.066) (42), and RNAhybrid (v2.1.2) (43). All parameters were set to default, and the intersecting RNAs were selected for further analysis.

### Co-expression analysis

The establishment of co-expression relationships between lncRNAs or circRNAs and mRNAs was based on the correlation between pairwise RNA expression levels. The correlation function in R package stats (v4.2.1) was used to calculate the correlation coefficients with the method set to “Pearson”. Correlation coefficients were separately calculated for lncRNAs and mRNAs, or circRNAs and mRNAs. Co-expression relationship pairs were selected based on a correlation coefficient ≥ 0.999 and a p-value ≤ 0.0001 for further analysis.

### Gene functional enrichment analysis

The functional roles of differentially expressed mRNAs, the target miRNAs of miRNAs, and the co-expression miRNAs of lncRNAs or circRNAs, were explored using the online tool, g:Profiler (44). The “organism” was set as *Sus scrofa*, the biological process of Gene Oncology and KEGG pathways were selected in “data source”, and the other parameters were set as default.

### Construction of RNA interaction network and identification of hub genes

The lncRNA-miRNA-mRNA network, circRNA-miRNA-mRNA network, and ceRNA network was constructed and visualized of in Cytoscape (v3.8.2) software (45). Subsequently, cytoHubba (v0.1) (46) app of Cytoscape was used to determine hub genes.

### RT-qPCR validation

Quantitative real-time polymerase chain reaction (RT-qPCR) was employed to corroborate the expression patterns of selected mRNA, miRNA, lncRNA, and circRNA candidates. For each specimen, 1 μg of total RNA underwent reverse transcription to synthesize complementary DNA (cDNA) in adherence to the guidelines provided by the First Strand cDNA Synthesis Kit (Catalog #FSK-101; TOYOBO, Tokyo, Japan). The ensuing RT-qPCR amplifications were facilitated using the SYBR Green Real-time PCR Master Mix (Catalog #11201ES03; Yeasen, Shanghai, China) and executed on the LightCycler480 II platform (Roche, Basel, Switzerland). Expression quantifications were derived employing the 2^−ΔΔCt^ methodology, and data are delineated in terms of log2 fold change for enhanced clarity and interpretation.

## Results

### Experimental design and analytical workflow

In a quest to dissect the comprehensive transcriptomic landscape of the target cells following PRRSV infection in piglets, we utilized an all-inclusive transcriptomics approach to scrutinize the mRNAs, miRNAs, circRNAs, and lncRNAs in PAMs upon PRRSV infection. On 3, 7, and 21 dpi, three piglets per group were euthanized to serve as biological replicates. After the removal of lungs, PAMs were isolated via lavage, and the extracted cells were counted to maintain a consistent cell population across all samples. Subsequent to total RNA extraction, we embarked on comprehensive transcriptome sequencing. By employing bioinformatics analyses, differentially expressed mRNAs (DE-mRNAs), miRNAs (DE-miRNAs), circRNAs (DE-circRNAs), and lncRNAs (DE-lncRNAs) were identified. A functional enrichment analysis was also conducted on DE-mRNAs and the potential target genes of differentially expressed ncRNAs (DE-ncRNAs).

Following this, a targeted prediction was executed for DE-miRNAs against mRNAs, lncRNAs, and circRNAs. These were then intersected with the identified DE-mRNAs, DE-lncRNAs, and DE-circRNAs in our study, yielding regulatory relationships comprising DE-miRNAs and DE-mRNAs, DE-lncRNAs and DE-miRNAs, as well as DE-circRNAs and DE-miRNAs.

**Figure 1:**
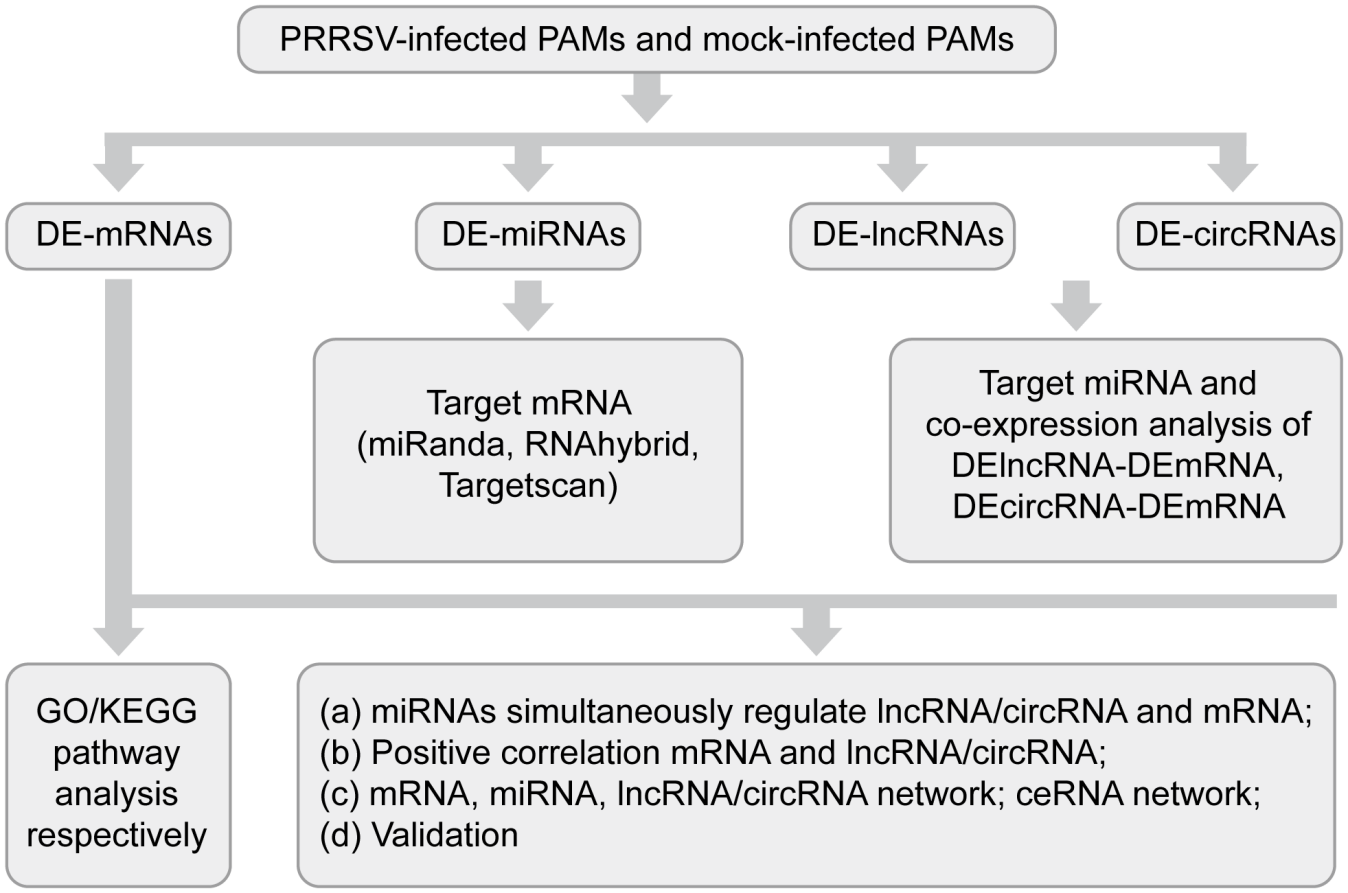
Workflow of integrated transcriptomic study.

Subsequently, correlation coefficients between lncRNAs or circRNAs and mRNAs were computed, resulting in the identification of co-expression pairs manifesting positive regulation: DE-lncRNAs and DE-mRNAs, DE-circRNAs and DE-mRNAs. An integrative analysis of these regulatory and positively co-expressed pairs led to the construction of the ceRNA network. To strengthen our findings, the transcriptome data was further corroborated by quantitative reverse transcription PCR (RT-qPCR) validation (**Figure 1**).

### Identification and quantification of differentially expressed RNAs

**Figure 2:**
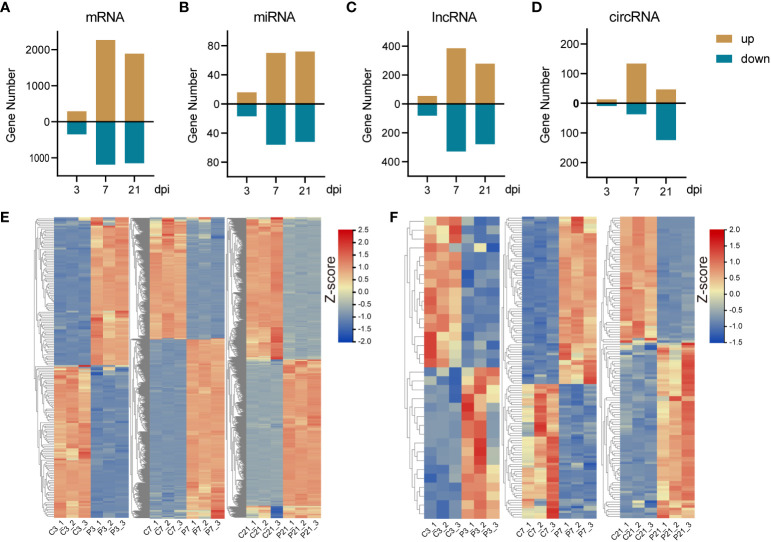
The summary of differentially expressed RNAs and expression pattern of DE-mRNAs and miRNAs. **(A-D)**, The bar plots of DE-mRNA, DE-miRNA, DE-lncRNA and DE-circRNA. **(E)** The heatmap of DE-mRNA at 3 (left panel), 7 (middle panel), and 21 (right panel) dpi. **(F)** The heatmap of DE-miRNA at 3 (left panel), 7 (middle panel), and 21 (right panel) dpi.

The principal component analysis (PCA) results demonstrate a high level of intragroup reproducibility and significant intergroup differences, indicating the feasibility of proceeding with further differential gene expression analysis (**Supplementary Figure 1**). DE-mRNAs, DE-lncRNAs, DE-miRNAs, and DE-circRNAs were discerned and sifted with a threshold of | log_2_(fold change) | ≥ 1 and p-adjust ≤ 0.05. This study revealed, at 3 dpi, a detection of 293 upregulated and 345 downregulated DE-mRNAs. A surge in DE-mRNAs was observed at 7 dpi, with 2269 upregulated and 1184 downregulated DE-mRNAs. This number tapered to 1888 upregulated and 1150 downregulated DE-mRNAs by 21 dpi (**Figures 2A, E**; **Supplementary Figures 3A–C**). As for DE-lncRNAs, the tally of upregulated DE-lncRNAs at 3, 7, and 21 dpi were 55, 385, and 279, respectively, whereas the downregulated DE-lncRNAs were identified as 81, 329, and 279, in that order (**Figure 2C**; **Supplementary Figures 2A**, **4A–C**). The quantity of DE-miRNAs was notably less. The numbers of upregulated DE-miRNAs at 3, 7, and 21 dpi were 16, 70, and 72, respectively, contrasted with 17, 56, and 52 downregulated DE-miRNAs at the respective timepoints (**Figures 2B, F**; **Supplementary Figures 3D–F**). Lastly, in the case of DE-circRNAs, the count of upregulated DE-circRNAs at 3, 7, and 21 dpi were 13, 134, and 47, respectively. Downregulated DE-circRNAs, on the other hand, were observed to be 9, 37, and 124, respectively (**Figure 2D**; **Supplementary Figures 2B**, **4D–F**). All DE-mRNA, DE-miRNA, DE-lncRNA and DE-circRNA were listed in the **Supplementary Tables 1**-**4**.

### Functional enrichment analysis of DE-mRNAs and target genes of DE-miRNAs

**Figure 3:**
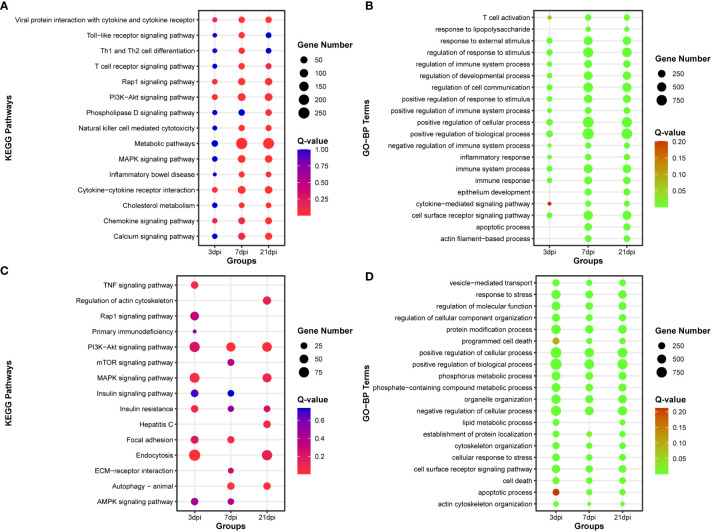
Functional enrichment analysis of DE-mRNA and target mRNA of DE-miRNA. **(A)** The top 15 KEGG pathways enriched with DE-mRNA. **(B)** The top 20 Gene Ontology of biological process (GO-BP) terms enriched with DE-mRNA. **(C)** The top 15 KEGG pathways enriched with target mRNA of DE-miRNA. **(D)** The top 20 Gene Ontology of biological process (GO-BP) terms enriched with target mRNA of DE-miRNA.

Functional enrichment analysis was conducted on DE-mRNAs, and the top 15 KEGG pathways and the top 20 Gene Ontology biological process (GO-BP) terms were enriched with dysregulated DE-mRNAs (**Figures 3A, B**). The results revealed that as the duration of infection increased, pathways associated with innate immunity, such as Toll-like receptor signaling pathway, T cell receptor signaling pathway, and Natural killer cell mediated cytotoxicity, were significantly enriched. Additionally, numerous immunity-related GO biological process terms were also enriched.

After simultaneous prediction of DE-miRNA target genes using Targetscan (47), miRanda (48), and RNAhybrid (49), functional enrichment analysis was carried out on the intersecting target genes predicted by these three softwares (**Supplementary Figure 5**, **Supplementary Table 5**). PI3K-Akt signaling pathway and Autophagy were significantly enriched in the top 15 KEGG pathways, and the top 20 GO-BP terms were associated with phosphorus and lipid metabolic process (**Figures 3C, D**).

### Functional enrichment analysis co-expressed mRNA of DE-lncRNA, and DE-circRNA

**Figure 4:**
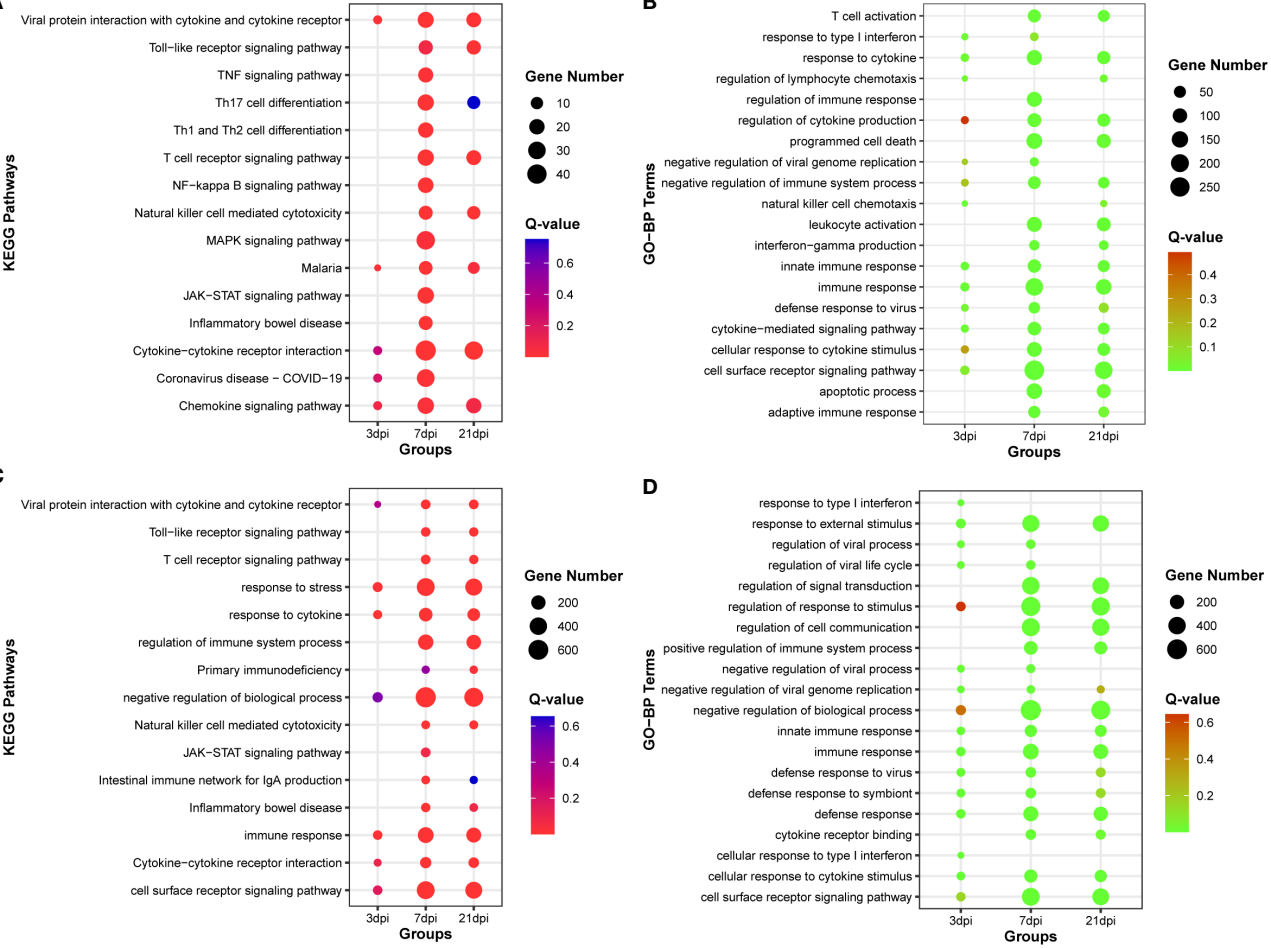
Functional enrichment analysis of co-expression mRNA of DE-lncRNA or DE-cirRNA. **(A)** The top 15 KEGG pathways enriched with co-expression mRNA of DE-lncRNA. **(B)** The top 20 Gene Ontology of biological process (GO-BP) terms enriched with co-expression mRNA of DE-lncRNA. **(C)** The top 15 KEGG pathways enriched with co-expression mRNA of DE-circRNA. **(D)** The top 20 Gene Ontology of biological process (GO-BP) terms enriched with co-expression mRNA of DE-circRNA.

By calculating the correlation coefficients between DE-circRNAs, DE-lncRNAs and DE-mRNAs, significant co-expression relationship pairs of mRNA-lncRNA and mRNA-CircRNA were selected based on r > 0.999 and p-value < 0.0001. The resulting co-expression target genes were subjected to enrichment analysis (**Supplementary Tables 8**-**9**). The top 15 KEGG pathways and the top 20 GO-BP terms by target genes co-expressed with DE-lncRNAs were enriched, respectively (**Figures 4A, B**). And the top 15 KEGG pathways and the top 20 GO-BP terms by target genes co-expressed with DE-circRNAs were also enriched, respectively (**Figures 4C, D**). The results indicate that genes associated with miRNAs, lncRNAs, and circRNAs are significantly enriched in innate immune regulation and resistance to viral invasion.

### The interplay of circRNA-miRNA-mRNA Network

**Figure 5:**
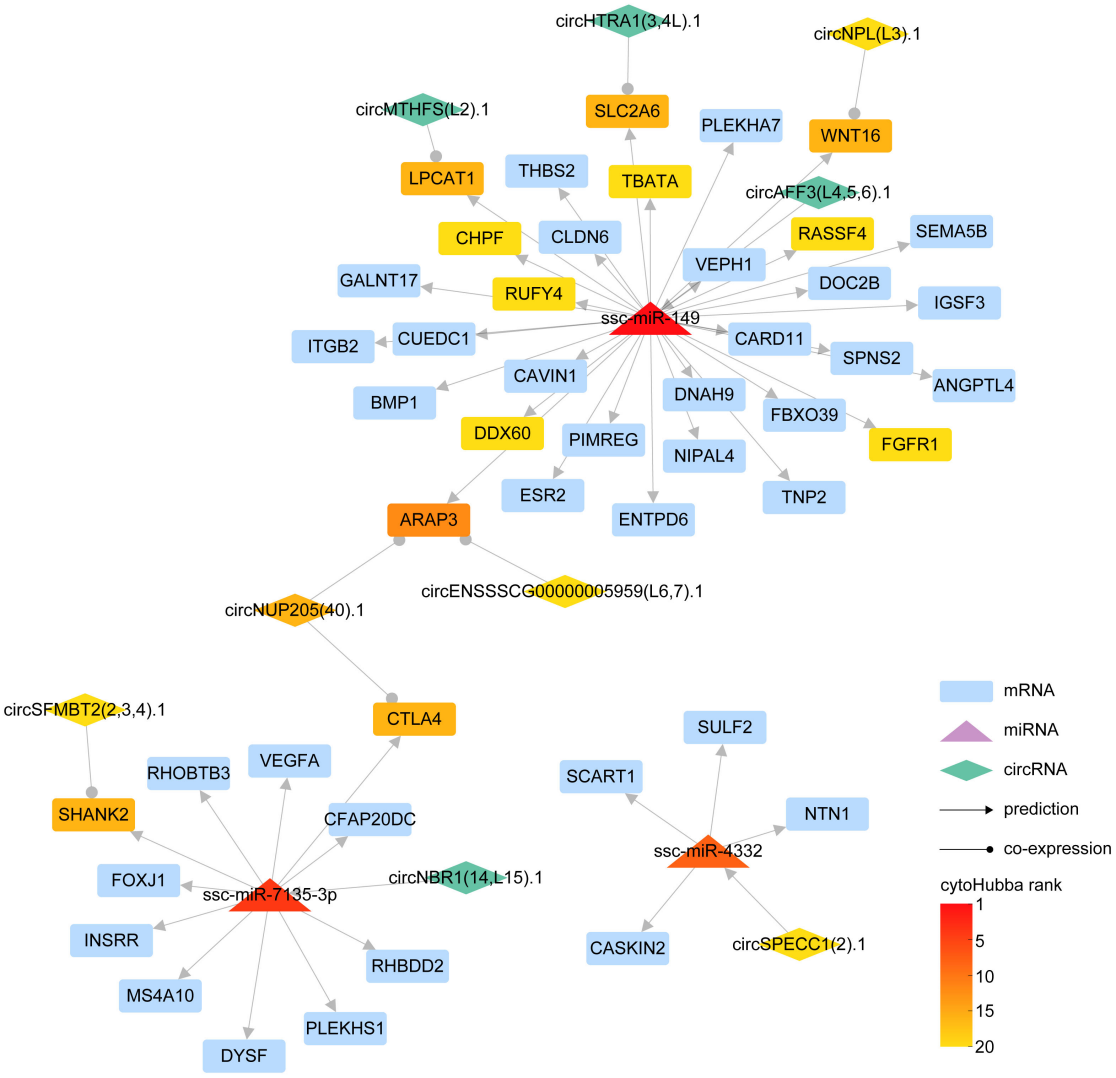
The interaction network of circRNA-miRNA-mRNA. In the schematic diagram, rectangles represent mRNAs, triangles denote miRNAs, and diamonds symbolize circRNAs. The lines adorned with arrowheads depict predicted interaction relationships, whereas those with circular markers represent co-expression relationships. The gradient of color signifies the ranking of different RNAs in cytoHubba.

MiRNAs exert a critical role in gene regulation by specifically binding to mRNAs 3’ UTR, inducing gene silencing and ultimately attenuating the expression of the target gene. Concurrently, circRNAs, functioning as miRNA sponges, have the potential to indirectly modulate mRNA expression levels. Target miRNA of DE-circRNA was predicted by RNAhybrid, miRanda, and Targetscan, then the intersecting miRNA was identified (**Supplementary Figure 7**, **Supplementary Table 7**). To delve into the interplay between miRNA-circRNA in orchestrating gene expression in the aftermath of PRRSV infection, we integrated interacting pairs of circRNA-miRNA, mRNA-miRNA, and the co-expression pairs of circRNA and mRNA in three time points, thereby constructing an intricate circRNA-miRNA-mRNA regulatory network (**Figure 5**).

Our results revealed that ssc-miR-149, ssc-miR-7135-3p, and ssc-miR-4332 could be pivotal miRNAs instrumental in regulating mRNA expression. Specifically, ssc-miR-149 demonstrated a potential to regulate 32 genes, inclusive of ITGB2, DDX60, and SLC2A6, with circRNA circAFF3(L4,5,6).1 exhibiting the capacity to influence the expression of these genes via ssc-miR-149. In parallel, ssc-miR-7135-3p displayed an ability to regulate the expression of 11 genes, for instance, CTLA4, SHANK2, and VEGFA, with circRNA circNBR1(14,L15).1 manipulating the expression of these genes through ssc-miR-7135-3p. Further, ssc-miR-4332 could modulate the expression of genes, including CASKIN2, NTN1, SULF2, and SCART1, with circRNA circSPECC1 (2).1 controlling the expression of these genes via ssc-miR-4332.

In essence, we succeeded in constructing a detailed network, mapping out the complex interplay between miRNA, which directly regulates gene expression, and circRNA, which modulates gene expression indirectly via miRNA.

### The interplay of lncRNA-miRNA-mRNA Network

**Figure 6:**
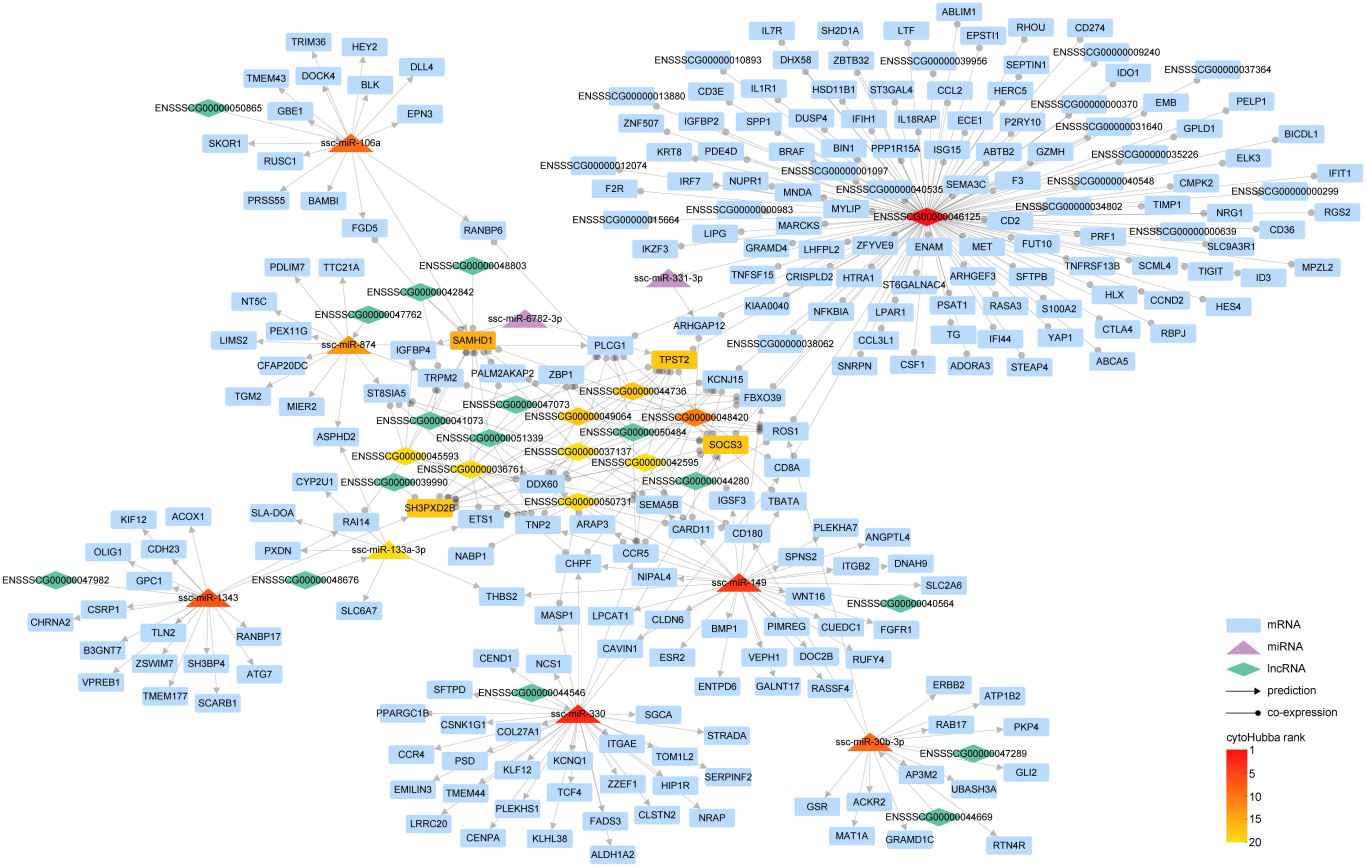
The interaction network of lncRNA-miRNA-mRNA. Within the graph, various entities are represented by distinct geometric shapes: mRNAs by rectangles, miRNAs by triangles, and lncRNAs by diamonds. Relationships derived from predictive analysis are denoted by lines marked with arrowheads, while co-expression relationships are illustrated by lines equipped with circular indicators. The gradation of color corresponds to the hierarchical positioning of different RNA types as determined by cytoHubba.

Similar to circRNA, lncRNA also serves as a sponge for miRNA, exerting an indirect influence on gene expression. Target miRNA of DE-lncRNA was predicted by RNAhybrid, miRanda, and Targetscan in the same way of circRNA targeted miRNA prediction, then the intersecting miRNA was discerned (**Supplementary Figure 6**, **Supplementary Table 6**).To unravel the intricate relationship between DE-miRNAs and DE-lncRNAs in modulating gene expression subsequent to PRRSV infection, we synthesized the interaction pairs of lncRNA-miRNA, mRNA-miRNA, and the co-expression pairs of lncRNA and mRNA, thereby forming a comprehensive lncRNA-miRNA-mRNA inter-regulatory network (**Figure 6**).

Our findings illustrate that a complex regulatory network was woven around key lncRNAs such as ENSSSCG00000046125, ENSSSCG00000048420, and ENSSSCG00000044736. Notably, ENSSSCG00000046125 displayed a co-expression relationship with 248 genes, including prominent ones such as ISG15, IL1R1, IFIH1, and IFIT1. This suggests that this lncRNA might orchestrate the expression of these genes via specific miRNAs. Analogous to circRNA circAFF3(L4,5,6).1, lncRNA ENSSSCG00000040564 has been found to regulate the expression of genes like SLC2A6 through ssc-miR-149. Furthermore, lncRNA ENSSSCG00000044546 could govern the expression of 33 genes via ssc-miR-330, and ENSSSCG00000047982 could modulate the expression of 18 genes via ssc-miR-1343.

In essence, we have meticulously constructed a network that encapsulates the complex interplay of miRNA directly governing gene expression and lncRNA modulating gene expression indirectly via miRNA.

### The competitive endogenous RNA network

**Figure 7:**
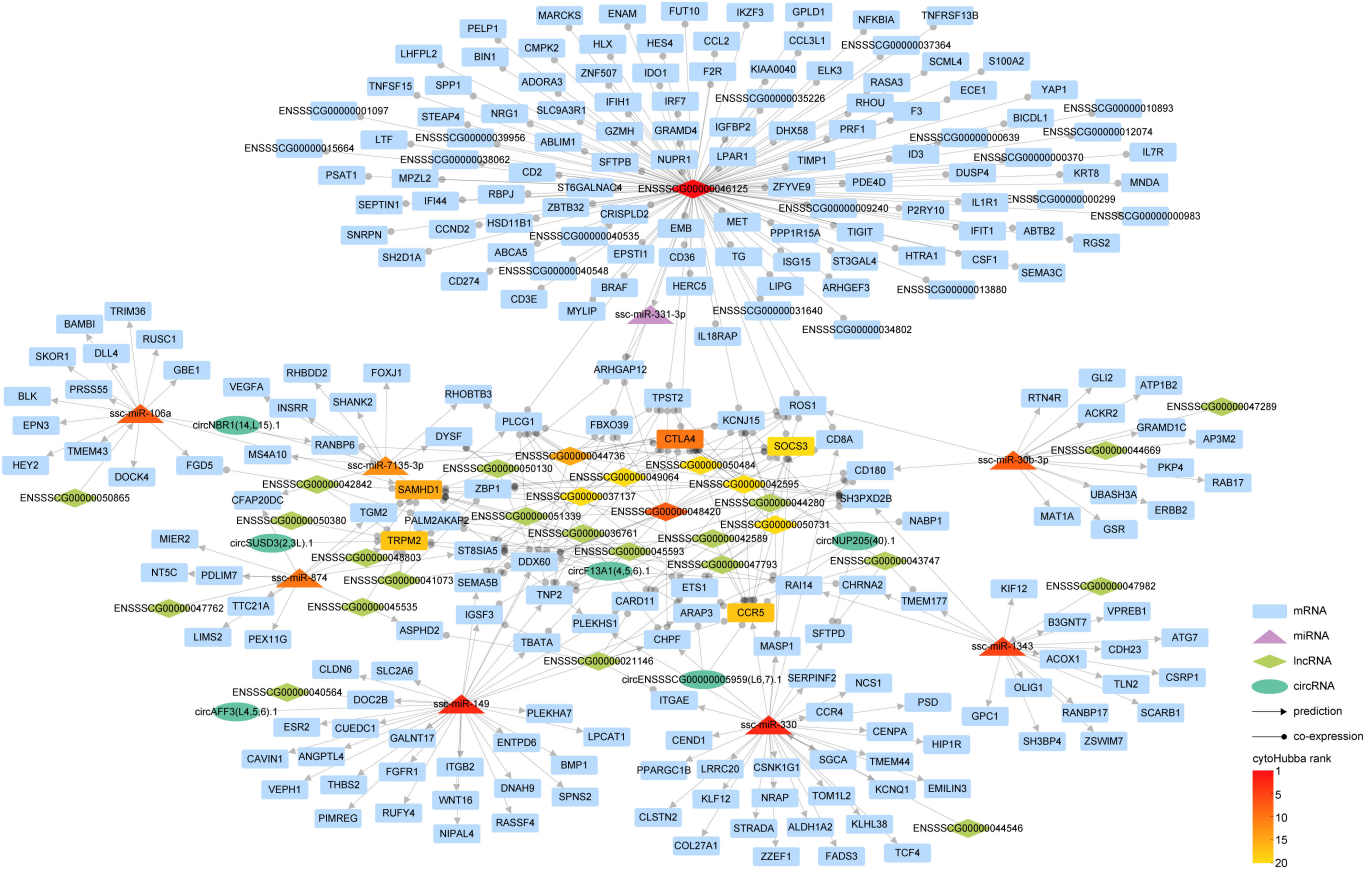
The interaction network of competitive endogenous RNA (ceRNA). In our graphical representation, distinct geometric forms correspond to different entities: rectangles for mRNAs, triangles for miRNAs, diamonds for lncRNAs, and ellipses for circRNAs. Interaction relationships inferred through predictive analyses are indicated by lines terminating with arrowheads, while co-expression correlations are symbolized by lines featuring circular markers. The gradated color scheme mirrors the hierarchical stratification of the various RNA types, as ranked by cytoHubba.

Within cellular biology, competitive endogenous RNAs (ceRNAs) constitute a diverse ecosystem, including mRNAs, miRNA, lncRNAs, and circRNAs. These ceRNAs engage in a dynamic interplay where they modulate one another’s expression levels by competitively binding the same miRNAs via miRNA response elements. To elucidate the interconnected roles of circRNAs and lncRNAs in controlling mRNA expression through miRNAs, we amalgamated interaction pairs of circRNA-miRNA, lncRNA-miRNA, mRNA-miRNA, alongside the co-expression pairs of either circRNA or lncRNA with mRNA, thereby constructing an intricate ceRNA regulatory network (**Figure 7**).

Our investigation has revealed an intriguing discovery regarding the integration of circRNA into the lncRNA-miRNA-mRNA interaction paradigm. This integration has significantly enriched the regulatory landscape of RNAs, shedding light on a more comprehensive understanding of gene expression regulation. During our analysis, we identified several pairs of ceRNAs. One such pair involves CTLA4, which is associated with the miRNA ssc-miRA-7135-3p, circRNA circNUP205 (40).1, and 11 lncRNAs, including ENSSSCG00000042595. Another pair consists of the lncRNA ENSSSCG00000040564 and circRNA circAFF3(L4,5,6).1, both competing for binding with ssc-miR-149, these ceRNA pairs exert regulatory control over the expression of specific genes, such as ITGB2.

This finding highlights the nuanced complexity within the ceRNA network, demonstrating the intricate interplay among circRNAs, lncRNAs, miRNAs, and mRNAs.

### Validation of transcriptomic data through RT-qPCR

**Figure 8:**
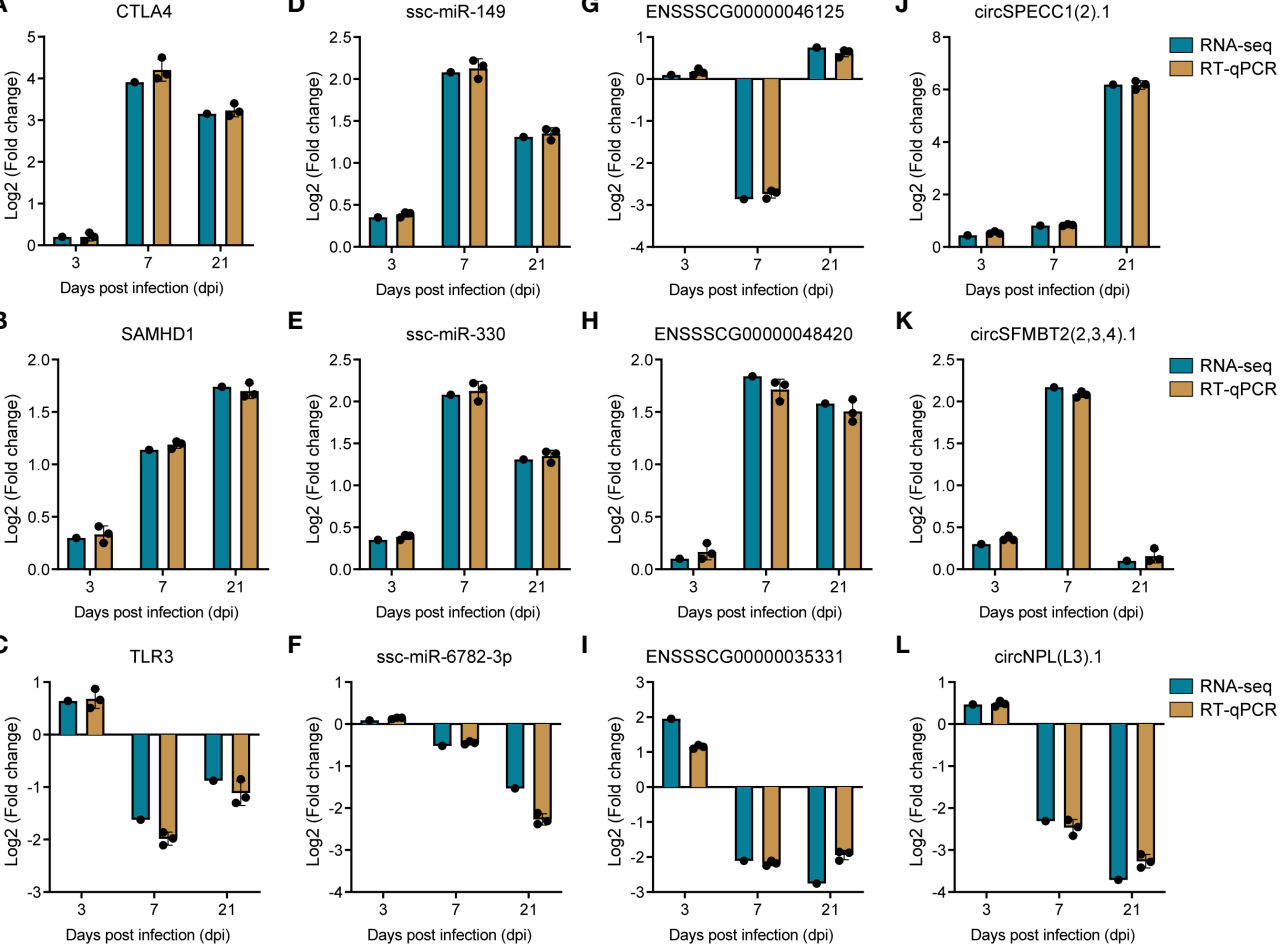
Validation of RNA sequencing data by RT-qPCR. **(A–C)** Barplots of fold change of mRNA CTLA4, SAMHD1 and TLR3. **(D–F)** Barplots of fold change of miRNA ssc-miR-149, ssc-miR-330, and ssc-miR-6782-3p. **(G–I)** Barplots of fold change of lncRNA ENSSSCG00000046125, ENSSSCG00000048420, and ENSSSCG00000035331. **(J–L)** Barplots of fold change of circRNA circSPECC1 (2).1, circSFMBT1 (2,3,4).1, and circNPL(L3).1.

To corroborate the precision of our transcriptomic data, we systematically selected genes with potential roles in PRRSV infection, drawing from the plug-in cytoHubba (46) in Cytoscape (45). This led to the identification of the top two ranked DE-circRNAs, specifically circSPECC1 (2).1 and circSFMBT2 (2,3,4).1, from the circRNA-miRNA-mRNA interaction network, along with the top two ranked lncRNAs, namely ENSSSCG00000046125 and ENSSSCG00000048420, from the lncRNA-miRNA-mRNA interaction network. Concurrently, we selected the leading two ranked miRNAs (ssc-miR-149 and ssc-miR-330) and mRNAs (CTLA4 and SAMHD1) from the ceRNA interaction network for validation of expression trends. To ensure a comprehensive assessment, we also sought to validate down-regulated genes. TLR3, recognized as the sensor of viral dsRNAs pivotal to antiviral processes, was deliberately selected. Subsequently, its co-expressed lncRNA counterpart, ENSSSCG00000035331, was also incorporated into our validation cohort. As for miRNA, we chose ssc-miR-6782-3p due to its targeting relationship with SAMHD1. Completing the validation set, the downregulated circNPL(L3).1, ranked within the top 20 in the circRNA-miRNA-mRNA network, was elected. This strategic identification was followed by the empirical verification of the expression levels of these selected RNAs through reverse transcription quantitative polymerase chain reaction (RT-qPCR). The resulting data demonstrated a high degree of congruity between the RT-qPCR outcomes and the transcriptomic sequencing results, thereby underpinning the reliability of our initial transcriptomic sequencing data (**Figure 8**).

## Discussion

PRRSV imposes a significant burden on the global swine industry, leading to substantial economic losses (50). The lack of effective prevention or treatment strategies underscores the need for extensive investigations into PRRSV pathogenesis for the discovery of novel vaccines and drugs (1, 5), for instance, targeting noncoding RNAs (51). However, previous research in the field has largely centered around limited RNA types and in vitro models, lacking a prolonged infection period (13, 26–32).

To address aforementioned limitations, we conducted a comprehensive in vivo transcriptomic analysis spanning 21 days, mirroring chronic PRRSV infections. This led to the identification of differentially expressed RNAs (DE-mRNAs, miRNAs, circRNAs, and lncRNAs) in PAMs from PRRSV-infected pigs at 3, 7, and 21 dpi. Our findings may provide a pioneering and robust basis for understanding PRRSV pathogenesis and emphasize the value of multi-RNA type studies in viral infections.

As pivotal elements within the ceRNA network, miRNAs serve as a critical nexus linking circRNAs or lncRNAs with mRNAs. Notably, ssc-miR-149 emerged as a core molecule within circRNA-miRNA-mRNA, lncRNA-miRNA-mRNA, and the broader ceRNA network (**Figures 5**–**7**) and was found upregulated at 7 and 21dpi (**Supplementary Table 2**), suggesting its potentially vital role during PRRSV infection. In a previous study, ssc-miR-149 has been confirmed to participate in porcine Sertoli cells functions by regulating tumor necrosis factor receptor (TNFR)-associated factor 3 (TRAF3) (52). Moreover, this miRNA, conserved between pigs and humans, has been widely reported to significantly contribute to the regulation of muscle growth and development, as well as porcine meat quality and precocious puberty (53, 54). Beyond those central miRNAs within the interaction network, ssc-miR-10b and ssc-miR-9-1 were observed to be upregulated at 3 dpi, and at 7 and 21 dpi, respectively. Both miRNAs are postulated to modify the distinct antiviral reactions triggered by various IFNs in PRRSV-infected PAMs (30). In our current study, ssc-miR-145-5p, which has been previously observed to induce alternative macrophage priming during PRRSV infection (26), exhibited a downregulated pattern at 7 dpi. Similarly, ssc-miR-125b, predicted to suppress PRRSV viral loads (26), was also noted to be downregulated at 3 and 21 dpi. These patterns of downregulation may signify a potential immunosuppression strategy employed by PRRSV.

Recent years have witnessed numerous investigations striving to decipher the intricate interplay between lncRNAs and gene expression in the context of PRRSV infection (13, 27, 29, 31, 33, 55). Predominantly employing PAMs as an in vitro infection model, these studies have highlighted that DE-lncRNAs participate in a range of cellular signaling pathways, including viral infection, immune response, NF-kappa B signaling, and toll-like receptor signaling (29, 31, 33, 55). The transcriptomic analysis of porcine endometrial epithelial cells further revealed the associations of lncRNAs with apoptosis-related genes (27), and another study on the transcriptome of porcine trophoblast cells suggested PRRSV-induced regulation of apoptosis genes via lncRNAs (13). These insights provide substantial evidence concerning the mechanisms underlying PRRSV’s vertical transmission and the manifestation of reproductive failure. In our study, we endeavored to discern consistent regulatory patterns by comparing the identified DE-lncRNAs with those in prior research. Intriguingly, we found that DE-lncRNA ENSSSCG00000035331, which exhibited an upregulation at 3 dpi and downregulation at 7 and 21 dpi in our data, consistently manifested a downregulation pattern in primary PAMs 24 hours post-PRRSV infection according to previous investigations (29). However, when we attempted to compare other DE-lncRNAs, we encountered challenges due to the variable methodologies employed for lncRNA annotation across different studies, leading to inconsistencies in lncRNA nomenclature (27, 29, 33). This ambiguity impeded our ability to conduct effective comparative studies, and it was similarly the case for circRNAs. This predicament underscores the need for standardized methodologies in future research to ensure comparability and consistency across studies. The situation highlights the pressing need for the establishment of standardized methodologies in future research, to promote comparability and enhance the robustness of analyses.

Cytotoxic T lymphocyte antigen 4 (CTLA4) serves as a key immune checkpoint that crucially modulates T cell activation, akin to the well-known PD-1 (56). The exploration of antibodies or inhibitors aimed at CTLA4 has shown encouraging results in cancer treatment (57). PRRSV, noted for its immunosuppressive character, has been implicated in the inadequate activation of T cells (58). In the present study, CTLA4 emerged as a top one ranking mRNA in ceRNA network (**Figure 7**), suggesting a significant role in PRRSV pathogenesis. Further, our data suggest that an upregulation of CTLA4, with a log2 fold change 3.9 at 7 and 3.2 at 21 dpi (**Supplementary Table 1**), may contribute to long-term immune suppression, as corroborated by 11 concurrently upregulated, co-expressed lncRNAs and one circRNA circNUP205 (40).1 (**Supplementary Tables 2**, **3**). An intriguing observation is the apex of symptoms in PRRSV-infected pigs at 7 dpi, followed by a slight attenuation in the upregulation fold change of CTLA4 by 21 dpi. This might be indicative of the commencement of recovery mechanisms in pigs from PRRS, suggesting a potential temporal modulation of these genes during the infection’s progression. These findings align with three other investigations, which report CTLA4 upregulation during PRRSV-1 or PRRSV-2 infection (59–61), lending further credence to the prolonged immune suppressive effect of PRRSV. We also observed the upregulation of another gene, SAMHD1 (SAM domain and HD domain-containing protein 1), known for its antiviral properties triggered by interferon, and its capacity to restrain the proliferation of numerous RNA or DNA viruses (62, 63). Notably, SAMHD1 has not been previously linked to PRRSV infection and emerged alongside CTLA4 as one of the top two hub mRNAs. This positions SAMHD1 as a significant player within the PRRSV interaction landscape. The increased expression of this gene at 7 and 21 dpi suggests an elevated innate immune response throughout the observed duration. In the ceRNA network, SAMHD1 regulation appears to involve ssc-miR-106a, circRNA circF13A1 (4,5,6).1, and 9 lncRNAs (**Figure 7**), hinting at a complex regulatory network. The elucidation of these two significant immune-related genes, CTLA4 and SAMHD1, and their associated miRNAs, lncRNAs, or circRNAs, highlights potential therapeutic targets for improved prevention and treatment of PRRSV.

In conclusion, our study conducted a comprehensive analysis of mRNA, miRNAs, circRNAs, and lncRNAs in PAMs from piglets infected with PRRSV over an extended elongated period. Notably, the integration of circRNA into the ceRNA network in this study marks a novel contribution to this field. The discernment of pivotal genes, such as CTLA4 and SAMHD1, in conjunction with their associated miRNAs, lncRNAs, and circRNAs, highlights promising therapeutic targets for PRRS. These findings provide a solid foundation for further investigations into the mechanisms of PRRSV and suggest potential avenues for prevention and treatment.

Furthermore, the identified RNAs provide insight into the multifaceted cellular responses during PRRSV infection. Delving deeper into whether these differentially expressed mRNAs, lncRNAs, circRNAs, and miRNAs impact PRRSV replication and host-virus interplay will be crucial, and future research shall focus on elucidating this mechanism. The elucidation of their functional significance could pave the way for the development of innovative therapeutic strategies against PRRSV, meeting the global urgency to combat viral threats.

## Data availability statement

The datasets presented in this study can be found in online repositories. The names of the repository/repositories and accession number(s) can be found below: PRJNA982972 (SRA).

## Ethics statement

The animal study was approved by Institutional Animal Care and Use Committee of Sun Yat-sen University. The study was conducted in accordance with the local legislation and institutional requirements.

## Author contributions

HZ: Conceptualization, Investigation, Project administration, Supervision, Writing – review & editing. OP: Data curation, Formal Analysis, Investigation, Methodology, Software, Writing – original draft. YX: Data curation, Formal Analysis, Investigation, Methodology, Software, Visualization, Writing – original draft. FH: Investigation, Methodology, Software, Writing – original draft, RG: Data curation, Methodology, Resources, Writing – original draft. YH: Data curation, Formal Analysis, Methodology, Software, Writing – original draft. SZ: Investigation, Methodology, Writing – original draft. GH: Data curation, Investigation, Resources, Software, Writing – original draft. QX: Methodology, Software, Validation, Writing – original draft. CZ: Data curation, Formal Analysis, Resources, Software, Writing – original draft, YW: Data curation, Formal Analysis, Investigation, Resources, Writing – original draft. YC: Conceptualization, Funding acquisition, Supervision, Writing – review & editing, Project administration.

## Supplementary material

Supplementary Figure 1 Principal component analysis (PCA) of three sequencing datasets. **(A)** PCA of mRNA and lncRNA sequencing dataset. **(B)** PCA of circRNA sequencing dataset. **(C)** PCA of miRNA sequencing dataset. C3, C7, and C21 represent the mock-infected groups at 3, 7, and 21 dpi, P3, P7, and P21 represent the PRRSV-infected groups at 3, 7, and 21 dpi.

Supplementary Figure 2 Expression pattern of DE-lncRNA and DE-circRNA. **(A)** The heatmap of DE-lncRNA at 3 (left panel), 7 (middle panel), and 21 (right panel) dpi. **(B)** The heatmap of DE-circRNA at 3 (left panel), 7 (middle panel), and 21 (right panel) dpi.

Supplementary Figure 3 The expression trend of DE-mRNA and DE-miRNA. A-C. Volcano plots of DE-mRNA at 3, 7, and 21 dpi. **(A–C)** Volcano plots of DE-miRNA at 3, 7, and 21 dpi.

Supplementary Figure 4 The expression trend of DE-lncRNA and DE-circRNA. A-C. Volcano plots of DE-lncRNA at 3, 7, and 21 dpi. **(A–C)** Volcano plots of DE-circRNA at 3, 7, and 21 dpi.

Supplementary Figure 5 The intersection analysis of target mRNA of DE-miRNA predicted by three softwares RNAhybrid, miRanda, and Targetscan. **(A)** The venn plot of target mRNA of DE-miRNA at 3 dpi predicted by three softwares. **(B)** The venn plot of target mRNA of DE-miRNA at 7 dpi predicted by three softwares. **(C)** The venn plot of target mRNA of DE-miRNA at 21 dpi predicted by three softwares.

Supplementary Figure 6 The intersection analysis of target miRNA of DE-lncRNA predicted by three softwares RNAhybrid, miRanda, and Targetscan. **(A)** The venn plot of target miRNA of DE-lncRNA at 3 dpi predicted by three softwares. **(B)** The venn plot of target miRNA of DE-lncRNA at 7 dpi predicted by three softwares. **(C)** The venn plot of target miRNA of DE-lncRNA at 21 dpi predicted by three softwares.

Supplementary Figure 7 The intersection analysis of target miRNA of DE-circRNA predicted by three softwares RNAhybrid, miRanda, and Targetscan. **(A)** The venn plot of target miRNA of DE-circRNA at 7 dpi predicted by three softwares. **(B)** The venn plot of target miRNA of DE-circRNA at 21 dpi predicted by three softwares.

Supplementary Table 1 Table of differentially expressed mRNA.

Supplementary Table 2 Table of differentially expressed miRNA.

Supplementary Table 3 Table of differentially expressed lncRNA.

Supplementary Table 4 Table of differentially expressed circRNA.

Supplementary Table 5 Table of target mRNA of DE-miRNA.

Supplementary Table 6 Table of target miRNA of DE-lncRNA.

Supplementary Table 7 Table of target miRNA of DE-circRNA.

Supplementary Table 8 Table of co-expression mRNA of DE-lncRNA

Supplementary Table 9 Table of co-expression mRNA of DE-circRNA
