# Supplementary figures and images for: Integrative transcriptomic profiling of mRNA, miRNA, circRNA, and lncRNA in alveolar macrophages isolated from PRRSV-infected porcine
Source: Front Immunol. 2023 Aug 24;14:1258778. doi: 10.3389/fimmu.2023.1258778 (PMC10491896; doi:10.3389/fimmu.2023.1258778)

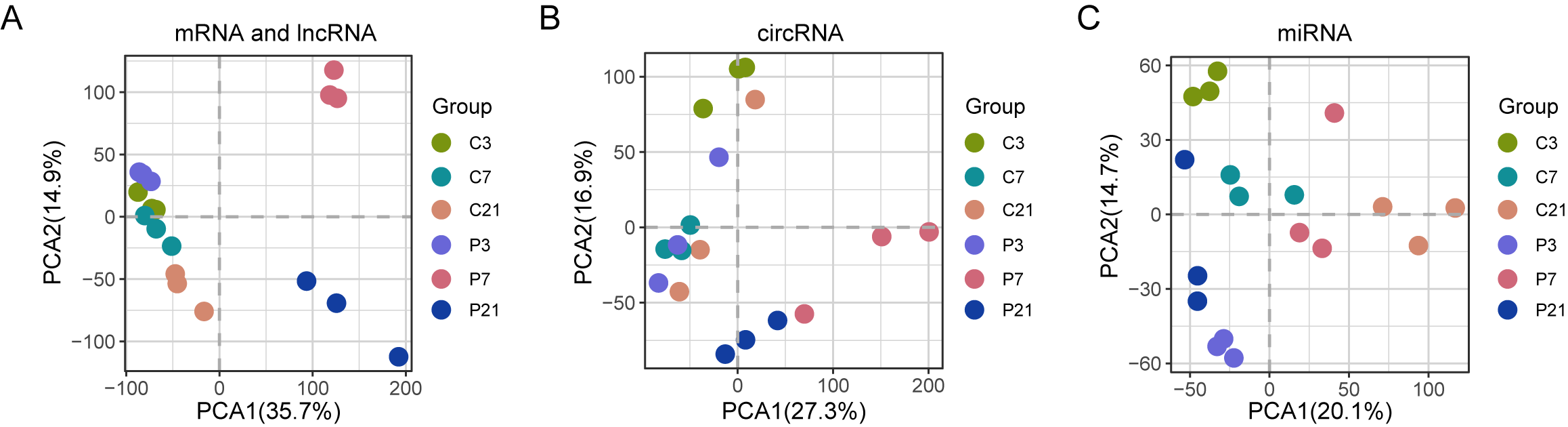

Supplement: Supplementary Figure 1 — Principal component analysis (PCA) of three sequencing datasets. (A) PCA of mRNA and lncRNA sequencing dataset. (B) PCA of circRNA sequencing dataset. (C) PCA of miRNA sequencing dataset. C3, C7, and C21 represent the mock-infected groups at 3, 7, and 21 dpi, P3, P7, and P21 represent the PRRSV-infected groups at 3, 7, and 21 dpi. [file Image_1.tif]

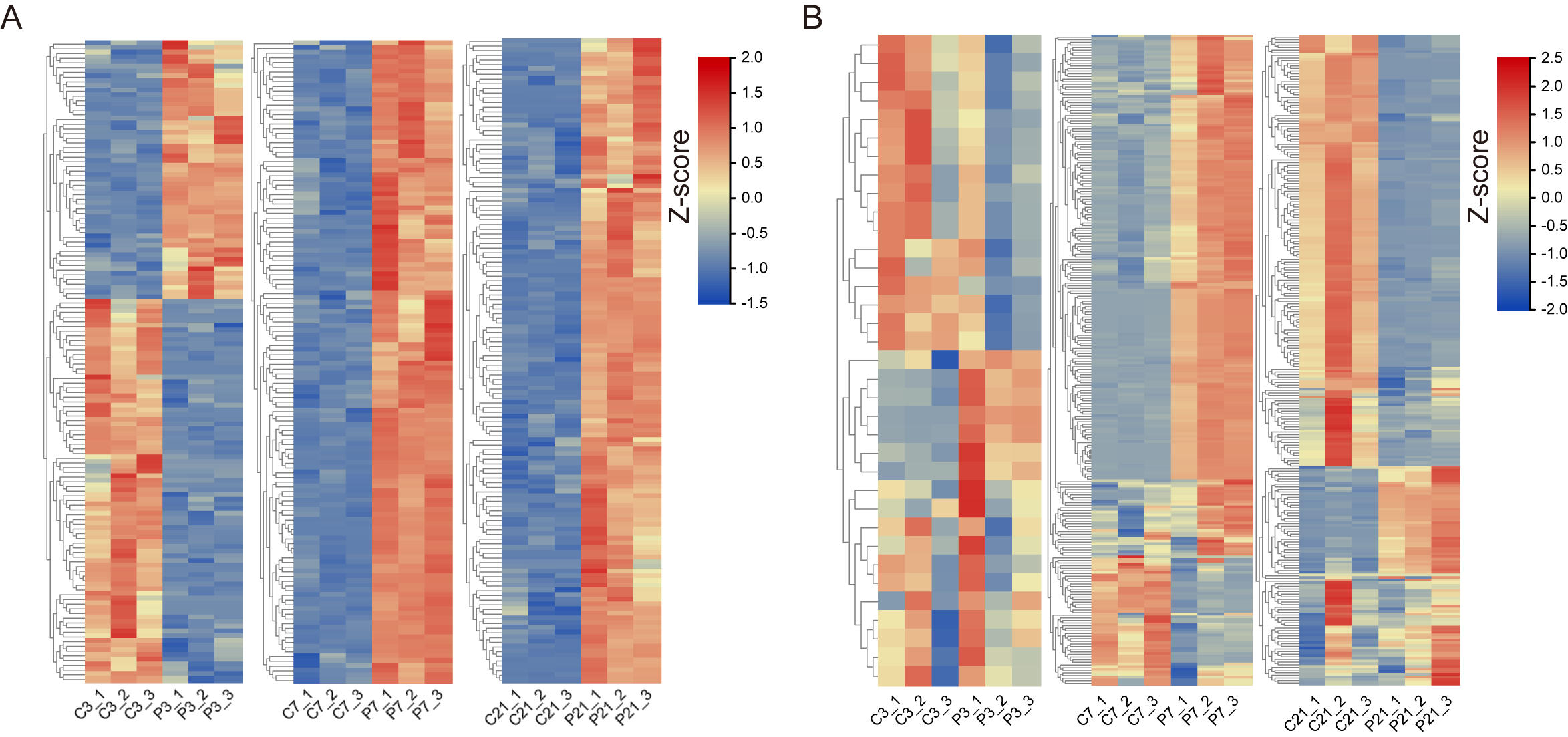

Supplement: Supplementary Figure 2 — Expression pattern of DE-lncRNA and DE-circRNA. (A) The heatmap of DE-lncRNA at 3 (left panel), 7 (middle panel), and 21 (right panel) dpi. (B) The heatmap of DE-circRNA at 3 (left panel), 7 (middle panel), and 21 (right panel) dpi. [file Image_2.tif]

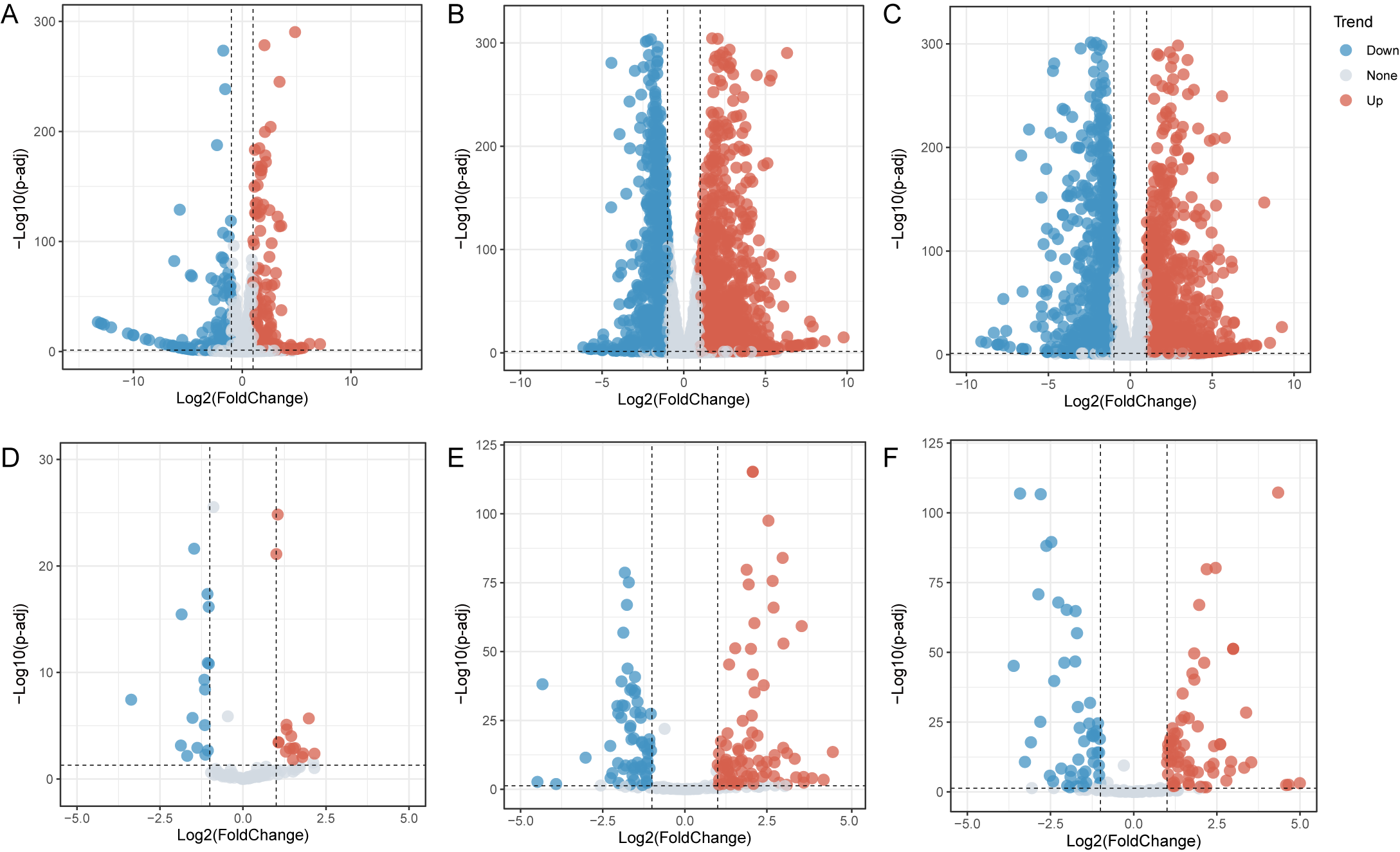

Supplement: Supplementary Figure 3 — The expression trend of DE-mRNA and DE-miRNA. A-C. Volcano plots of DE-mRNA at 3, 7, and 21 dpi. (A–C) Volcano plots of DE-miRNA at 3, 7, and 21 dpi. [file Image_3.tif]

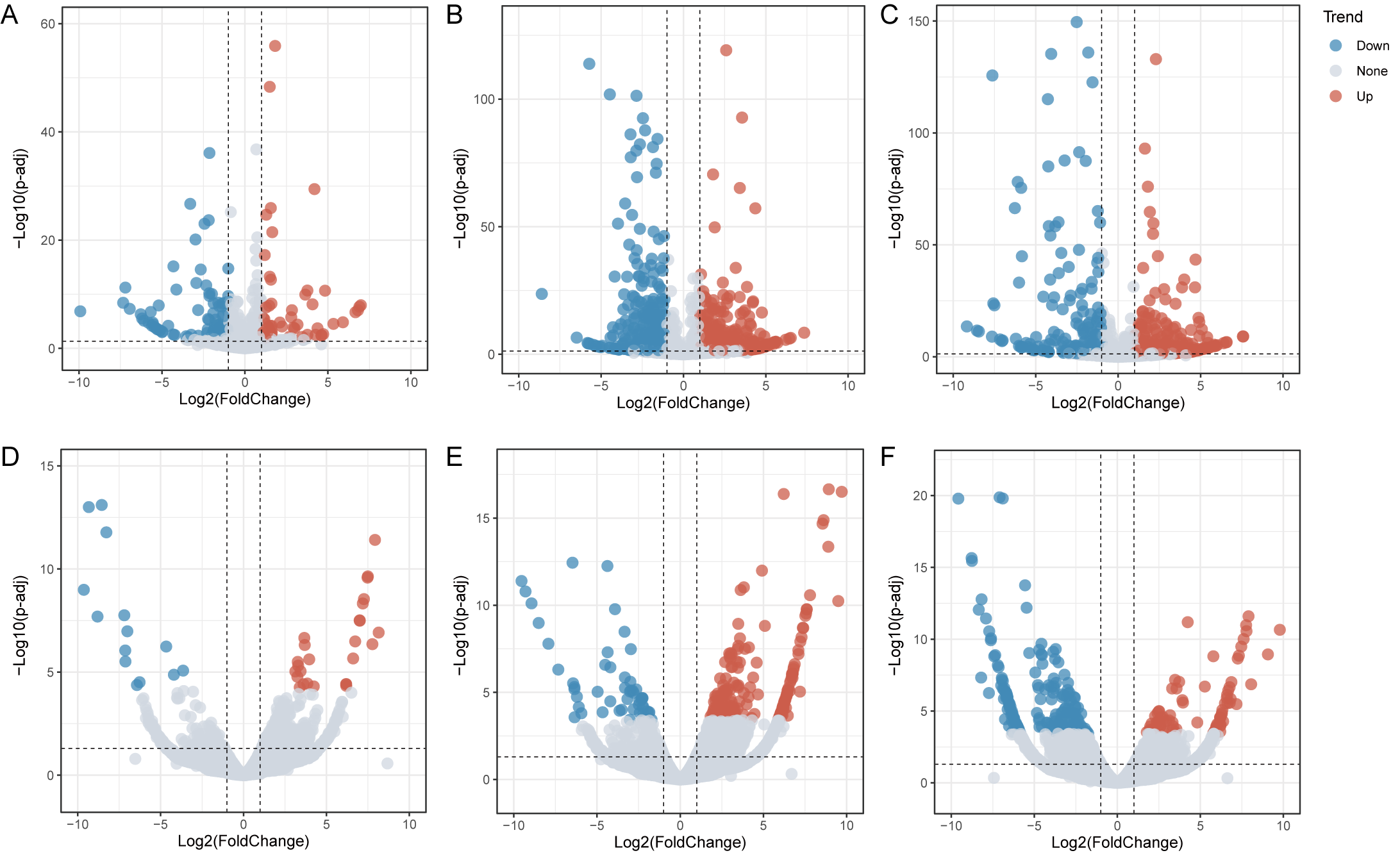

Supplement: Supplementary Figure 4 — The expression trend of DE-lncRNA and DE-circRNA. A-C. Volcano plots of DE-lncRNA at 3, 7, and 21 dpi. (A–C) Volcano plots of DE-circRNA at 3, 7, and 21 dpi. [file Image_4.tif]

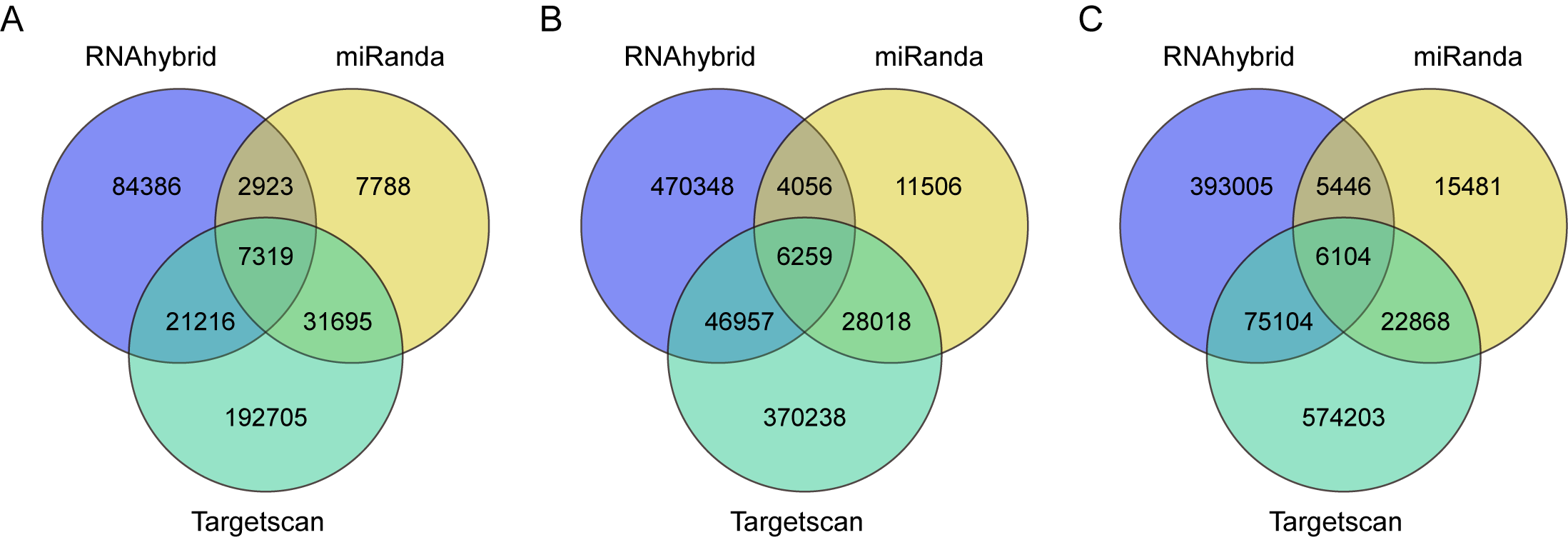

Supplement: Supplementary Figure 5 — The intersection analysis of target mRNA of DE-miRNA predicted by three softwares RNAhybrid, miRanda, and Targetscan. (A) The venn plot of target mRNA of DE-miRNA at 3 dpi predicted by three softwares. (B) The venn plot of target mRNA of DE-miRNA at 7 dpi predicted by three softwares. (C) The venn plot of target mRNA of DE-miRNA at 21 dpi predicted by three softwares. [file Image_5.tif]

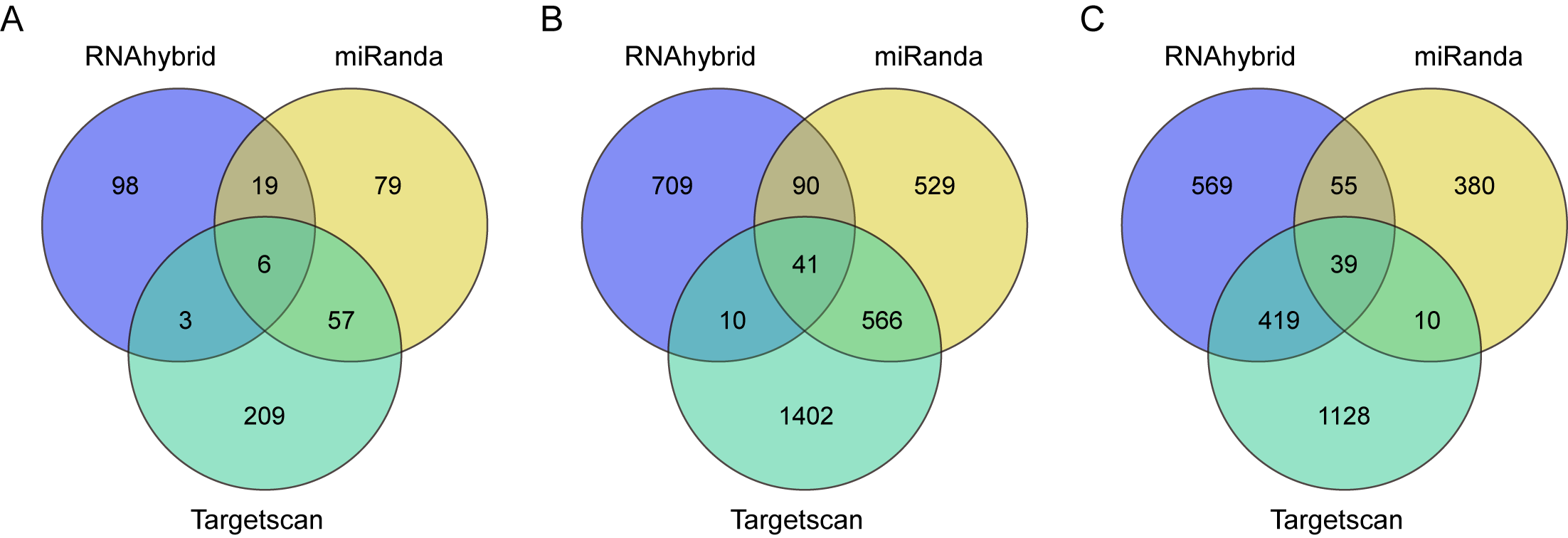

Supplement: Supplementary Figure 6 — The intersection analysis of target miRNA of DE-lncRNA predicted by three softwares RNAhybrid, miRanda, and Targetscan. (A) The venn plot of target miRNA of DE-lncRNA at 3 dpi predicted by three softwares. (B) The venn plot of target miRNA of DE-lncRNA at 7 dpi predicted by three softwares. (C) The venn plot of target miRNA of DE-lncRNA at 21 dpi predicted by three softwares. [file Image_6.tif]

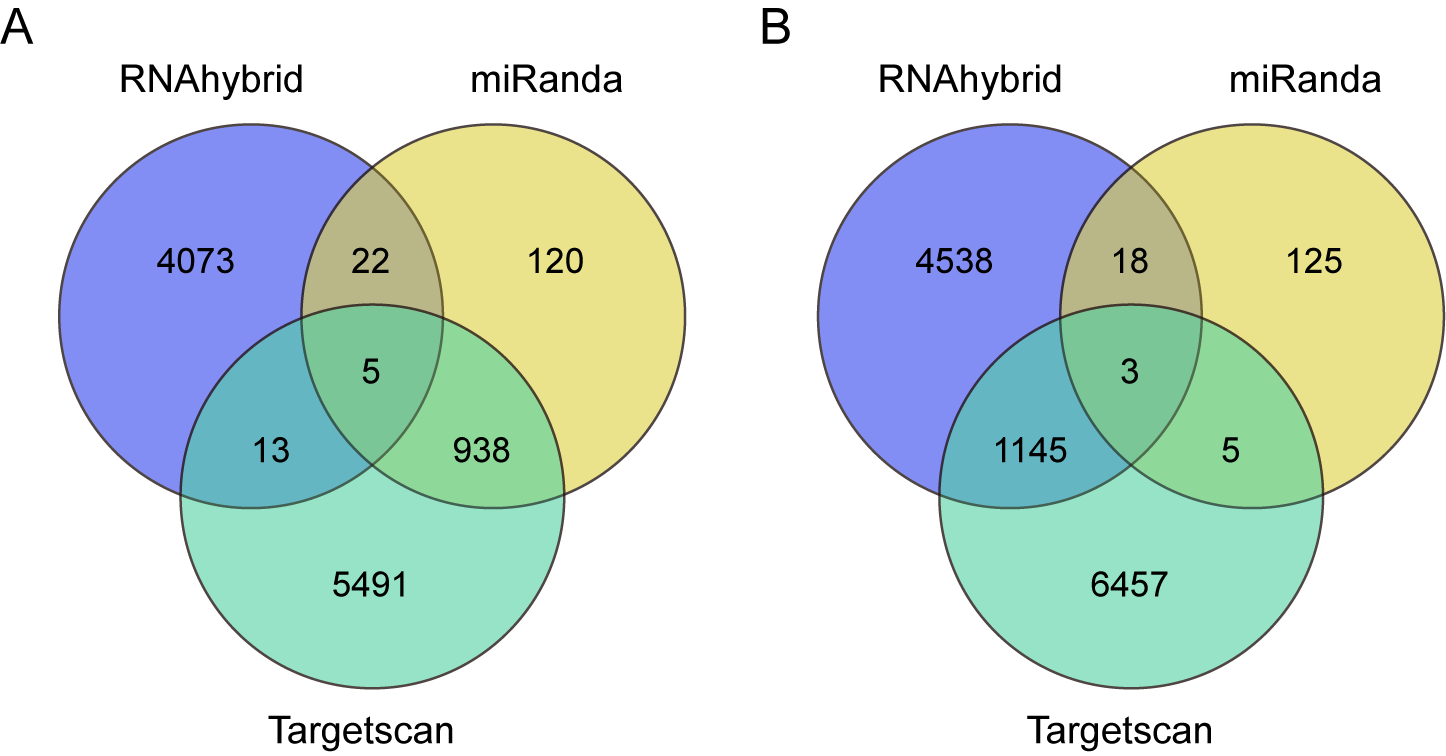

Supplement: Supplementary Figure 7 — The intersection analysis of target miRNA of DE-circRNA predicted by three softwares RNAhybrid, miRanda, and Targetscan. (A) The venn plot of target miRNA of DE-circRNA at 7 dpi predicted by three softwares. (B) The venn plot of target miRNA of DE-circRNA at 21 dpi predicted by three softwares. [file Image_7.tif]
